# Supplementary material for: In Silico Study of Camptothecin-Based Pro-Drugs Binding to Human Carboxylesterase 2
Source: Biomolecules. 2024 Jan 27;14(2):153. doi: 10.3390/biom14020153 (PMC10886758; doi:10.3390/biom14020153)
Supplement: Supplementary file 1 [file biomolecules-14-00153-s001.zip › beierlein_SI.R2.pdf]

Supplementary Information for In Silico Study of Camptothecin-Based Pro-drugs Binding to Human Carboxylesterase 2

Frank Beierlein[1,2], Anselm H. C. Horn[2,3], Heinrich Sticht[3], Andriy Mokhir[4], and Petra Imhof[1]

[1] Department for Chemistry and Pharmacy
Computer Chemistry Center
Friedrich-Alexander University (FAU) Erlangen Nürnberg
Nägelsbachstraße 25
91052 Erlangen
Germany

[2]Erlangen National High Performance Computing Center (NHR@FAU)
Friedrich-Alexander University (FAU) Erlangen Nürnberg
Martensstraße 1
91058 Erlangen
Germany

[3]Institute of Biochemistry
Friedrich-Alexander University (FAU) Erlangen Nürnberg
Fahrstraße 17
91054 Erlangen
Germany

[4]Department for Chemistry and Pharmacy
Institute for Organic Chemistry
Friedrich-Alexander University (FAU) Erlangen Nürnberg
Nikolaus-Fiebiger-Straße 10
91058 Erlangen
Germany

1.1 Protein

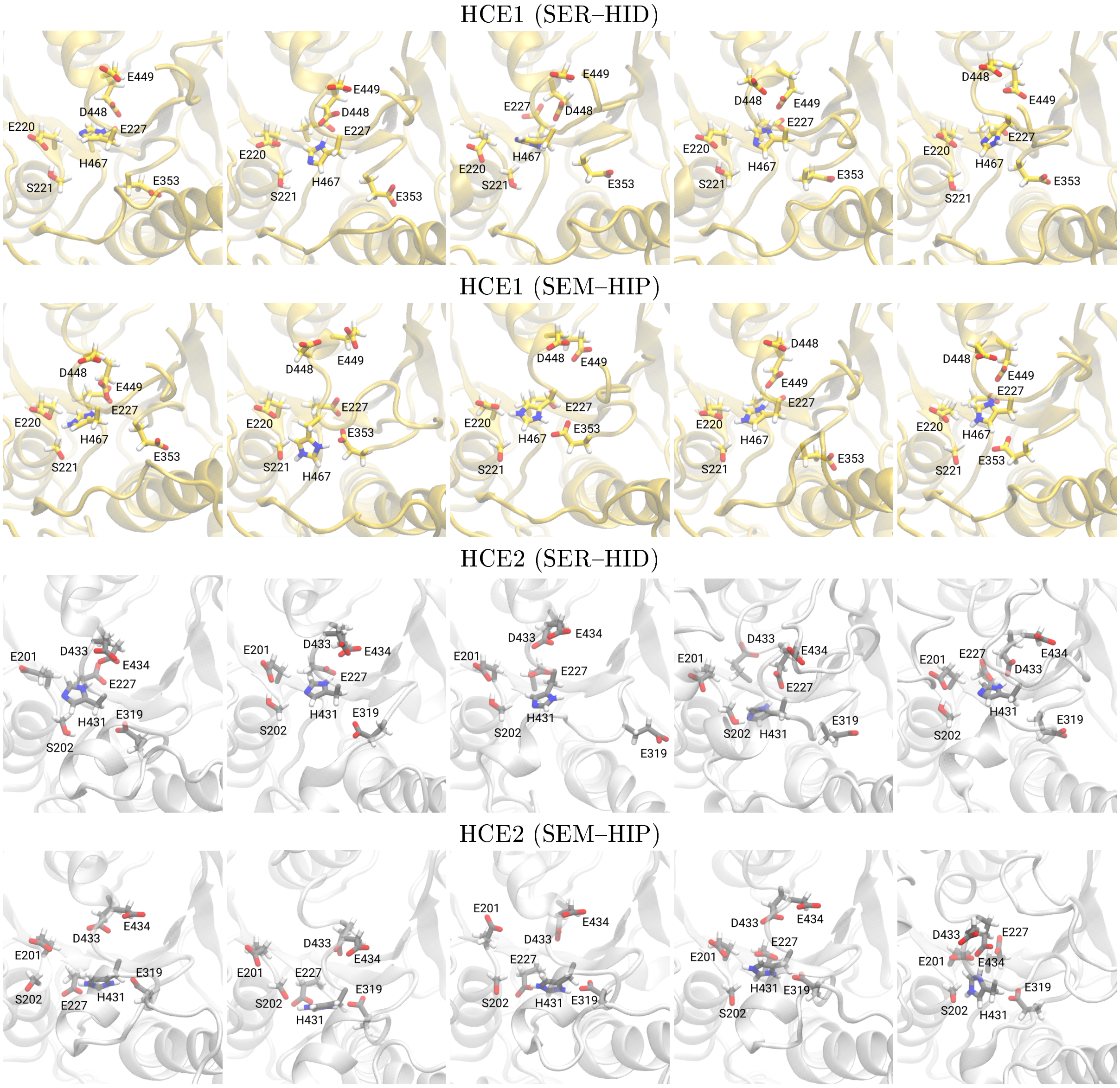

Figure S1: Snapshots (medoid structures) of the active site of the apo proteins HCE1 and HCE2 from all respective five simulation runs in the two different protonation states: neutral Ser and His (SER–HID) and deprotonated Ser, protonated His (SEM–HIP).

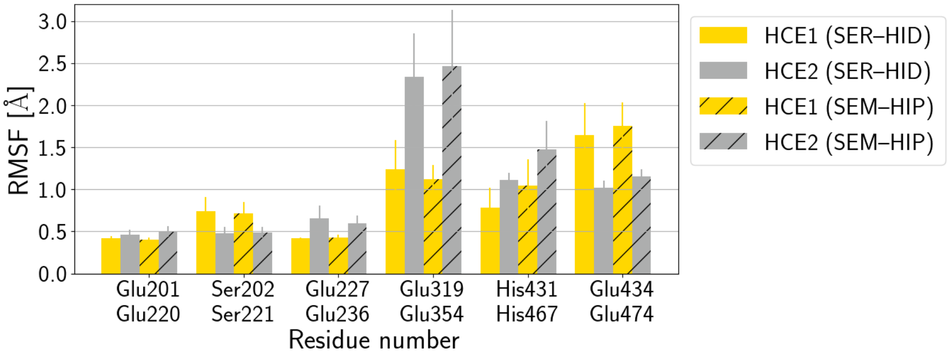

Figure S2: Root mean square fluctuations (RMSF) of selected residues in the apo proteins HCE1 and HCE2.

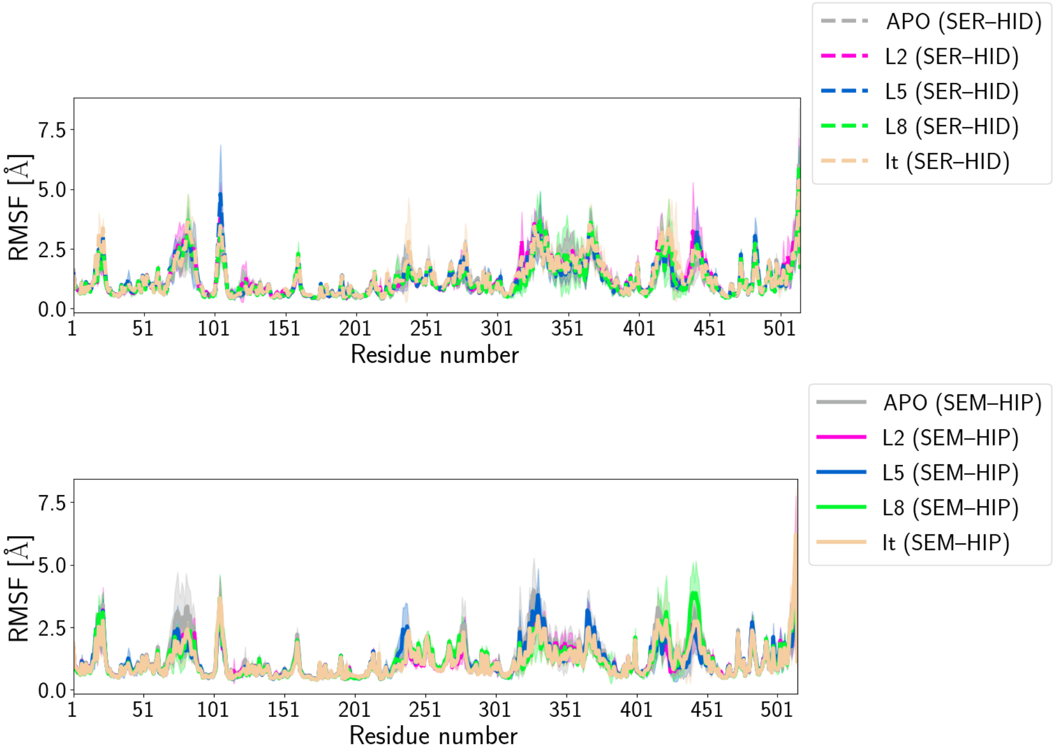

Figure S3: Root mean square fluctuations (RMSF) of the protein residues in HCE2 in apo form and bound to the ligands.

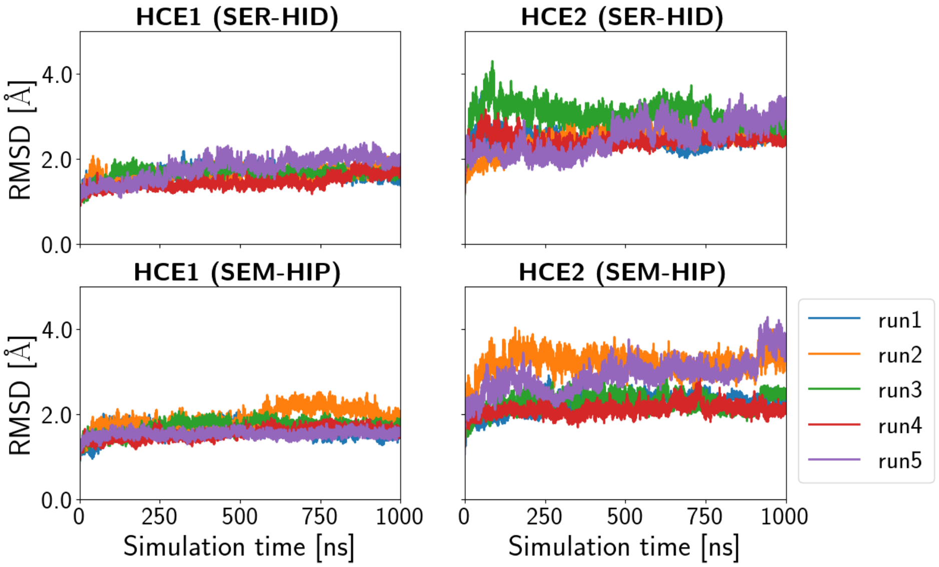

Figure S4: Time series of root mean square deviation (RMSD) in the course of the five independent simulation runs of the apo protein, HCE1 and HCE2, respectively.

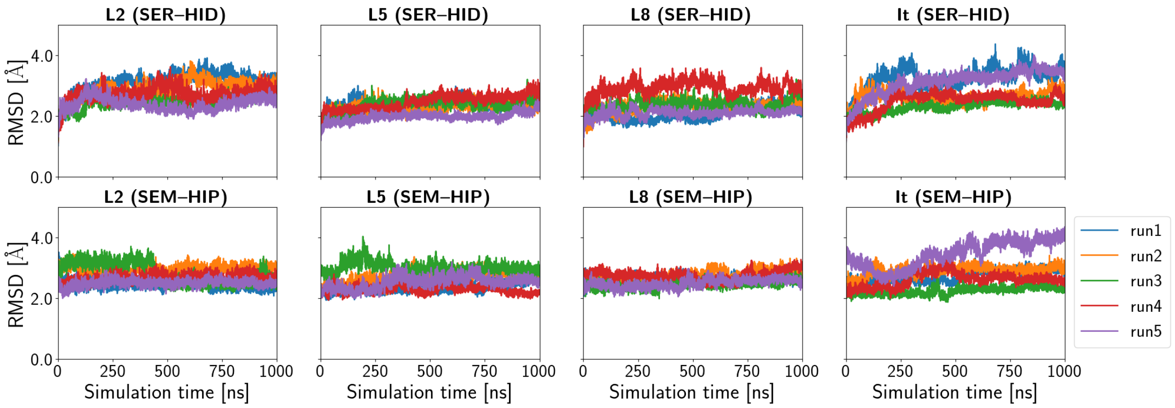

Figure S5: Time series of root mean square deviation (RMSD) in the course of the five independent simulation runs of the HCE2-ligand complexes.

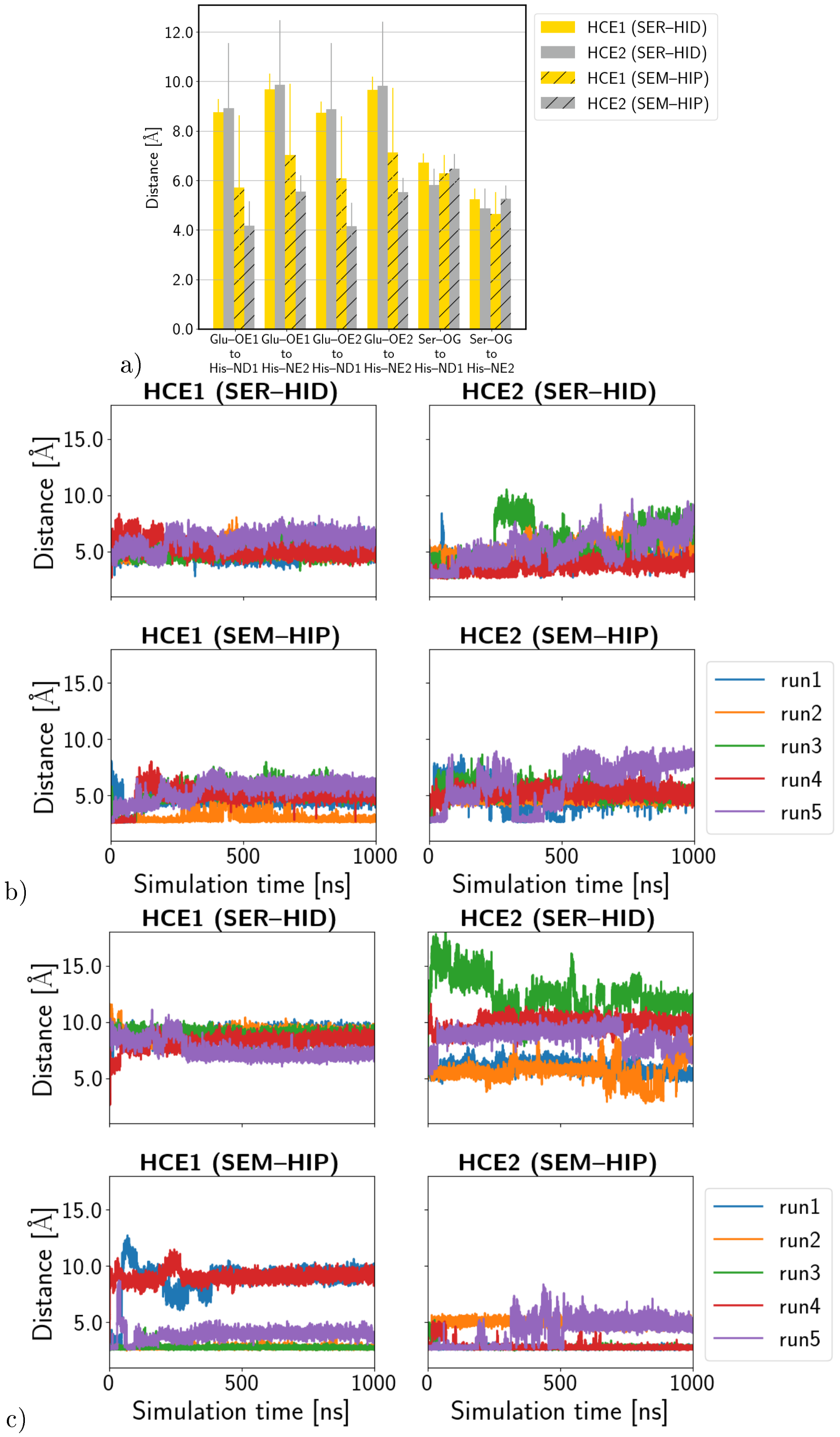

Figure S6: a) Average distances between residues of the catalytic triad in the apo proteins HCE2 and HCE1, b) time series of the distance between the Ser202/Ser221 oxygen atoms and the closest Glu319/Glu354 carboxyl oxygen atom and c) and time series of the distance between the Ser202/Ser221 oxygen atoms and the closest His431/His467 nitrogen atom in the apo proteins HCE2 and HCE1, respectively.

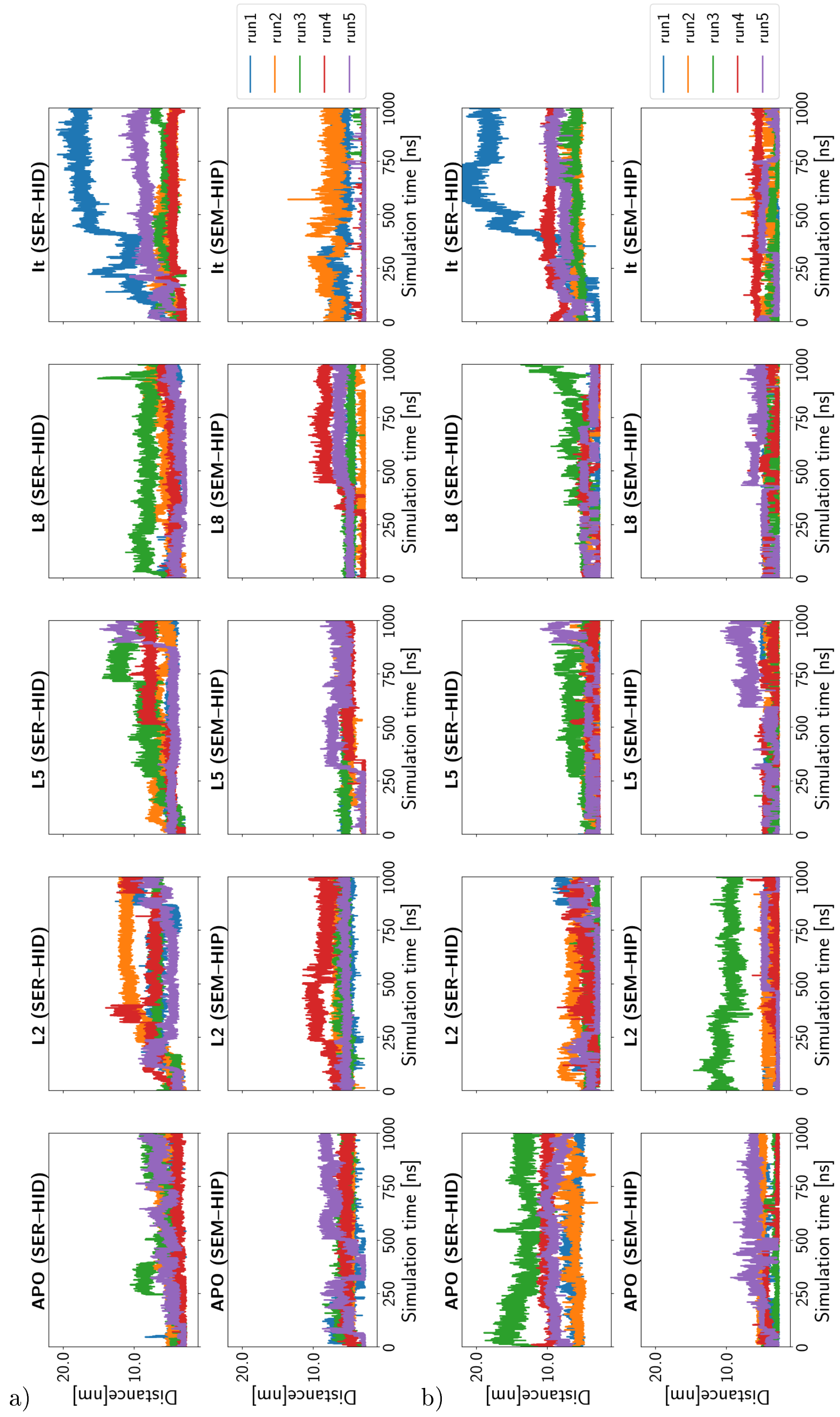

Figure S7: Time series of distances a) between Ser202–OG and His431–NE2 and b) between Glu319–OE2 and His431–NE2 in the models of HCE2.

1.2 Ligand conformations

The pro-drug ligands with different linker lengths, L2, L5, and L8, exhibit different folding conformations, as quantified by the distance between the ester C-atom and the nitrogen atom of the amino group, that can be directly related to the length of their linkers (Figure S8). Pro-drugs with a linker length of $n = 2$ show a narrow distribution of rather short (~7Å) C–N distances, whereas the pro-drugs with longer linker length $n = 5, 8$ show predominantly larger (~10Å and ~13Å, respectively) C–N distances in water. However, even with long linker length, also folded conformations are observed, corresponding to a smaller peak at ~5Å in the probability distributions of the C–N distances. For Irinotecan, which has no flexible linker, the protonated N-atom and the ester C-atom show a distance of ~6Å throughout. This distance is also kept in Irinotecan bound to the esterase protein. The other ligands, L2. L5, and L8, however, exhibit slightly "stretched" , but certainly no completely folded conformations, with C–N distances of ~10Å, when bound to the protein. This indicates that contacts with the protein overcompensate the intramolecular interactions in the L_n pro-drugs (between amino group and ester and/or lactone moiety and pi-stacking between the triazole ring and the camptothecin ABCD ring system).

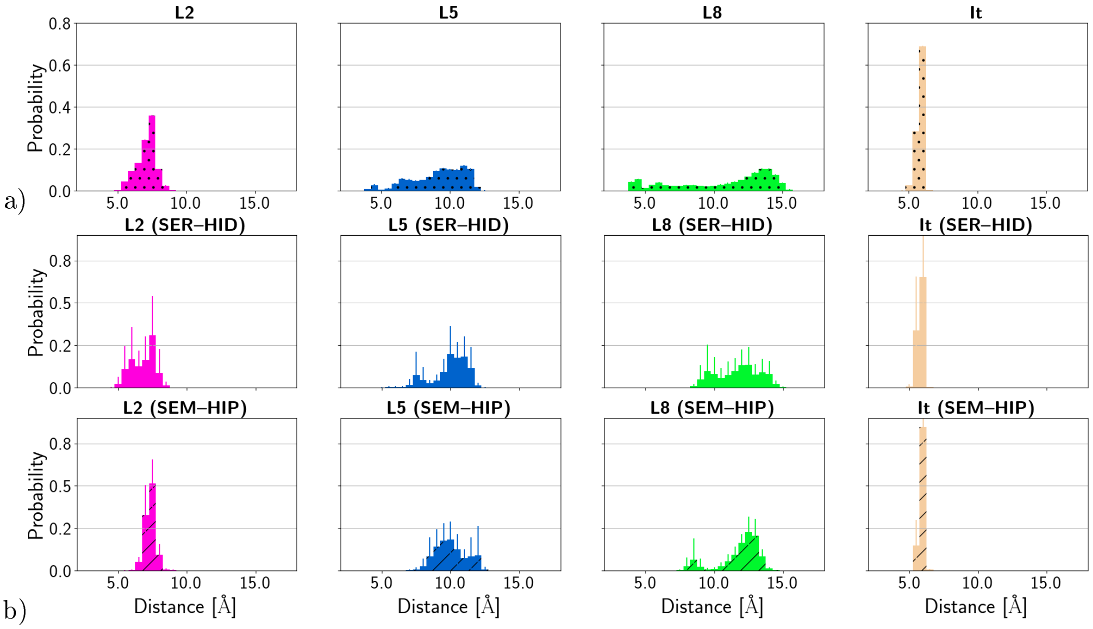

Figure S8: Probability distribution of the distance between the ester C and the N-atom of the protonated amino group (tertiary amino group in case of irinotecan) of the ligand a) in water and b) bound to the esterase protein HCE2.

The fluctuations of the ligand atoms (measured as root mean squared fluctuations) in water has a clear trend in which the largest ligand, L8, fluctuates more than the other ones and Irinotecan with its rather rigid scaffold fluctuates least. When bound to the protein, the overall fluctuations are, within error, comparable between the different ligands, indicating that the ligands all fluctuate similarly in their position in the binding pocket (rather than folding/unfolding).

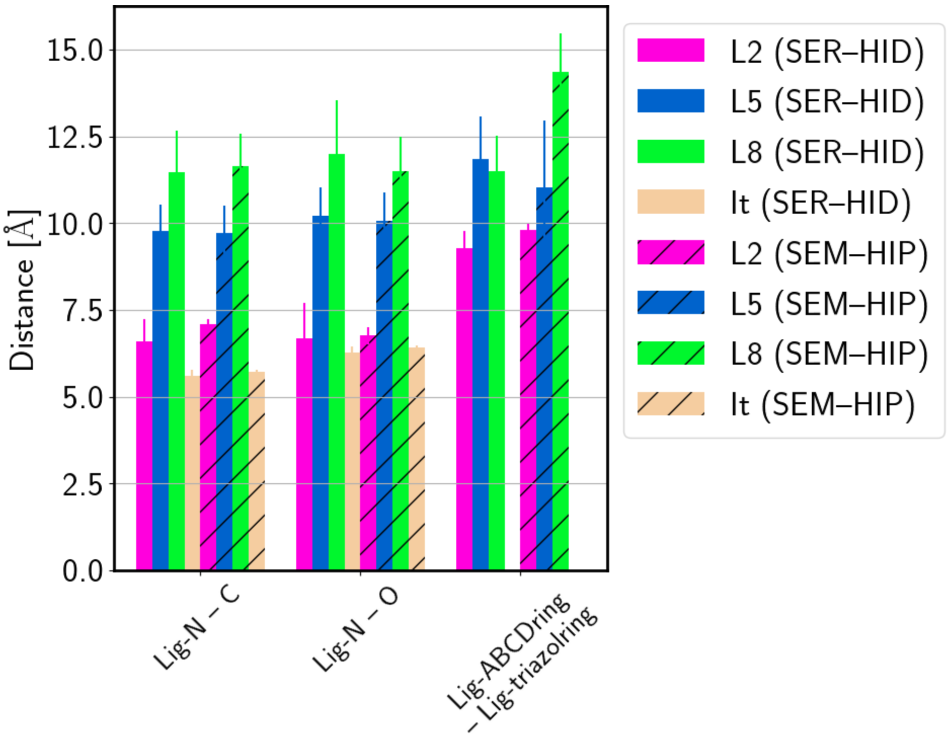

Figure S9: Intramolecular distances between parts of the ligands.

a) 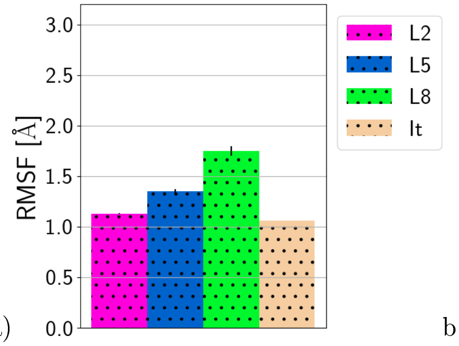

b) 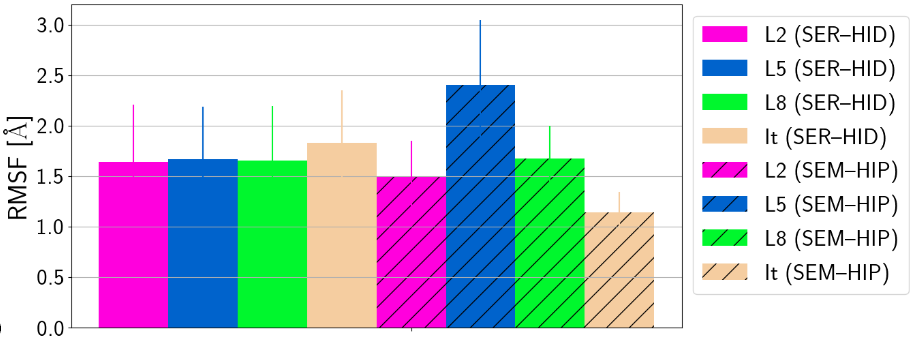

Figure S10: Root mean square fluctuations of the ligands a) in water and b) in the HCE2 protein.

Table S1: Shortest distances between Ser202–OG atom and the ester carbonyl atom of the ligand in the docked poses.

	distance [Å]
L2	4.31
L5	3.72
L8	3.53
It	2.64

1.3 Protein-Ligand interactions

Table S2: Van der Waals interaction energies [kcal/mol] between residues of the binding pocket and the different ligands.

(SER-HID)							
	Glu201	Ser202	Glu227	Glu319	His431	Asp433	Glu434
L2	-0.52 ± 0.95	-2.19 ± 0.79	0.16 ± 0.32	-0.20 ± 0.03	-2.77 ± 0.91	-0.15 ± 0.09	0.44 ± 0.60
L5	-2.06 ± 1.17	-2.37 ± 0.34	0.04 ± 0.23	-0.43 ± 0.22	-4.11 ± 0.50	-0.26 ± 0.08	0.80 ± 0.59
L8	-2.74 ± 1.66	-2.52 ± 0.65	0.32 ± 0.50	-0.50 ± 0.73	-4.22 ± 1.09	-0.24 ± 0.14	0.53 ± 0.67
It	-2.15 ± 0.69	-2.08 ± 0.87	-0.28 ± 0.25	-0.22 ± 0.19	-2.25 ± 1.03	-0.22 ± 0.13	-0.37 ± 0.32

(SEM-HIP)							
	Glu201	Ser202	Glu227	Glu319	His431	Asp433	Glu434
L2	-1.32 ± 0.43	-1.69 ± 0.36	0.84 ± 0.18	-0.34 ± 0.35	-3.91 ± 0.61	-0.32 ± 0.18	0.32 ± 0.11
L5	-1.39 ± 0.98	-1.74 ± 0.60	0.37 ± 0.38	-0.19 ± 0.25	-3.21 ± 1.10	-0.20 ± 0.06	0.49 ± 0.30
L8	-2.32 ± 1.00	-2.12 ± 0.95	0.21 ± 0.65	-0.32 ± 0.14	-3.55 ± 1.44	-0.29 ± 0.14	0.27 ± 0.21
It	-2.50 ± 0.47	-2.46 ± 0.76	-0.42 ± 0.56	-0.14 ± 0.14	-2.46 ± 2.15	-1.40 ± 1.04	-0.22 ± 0.05

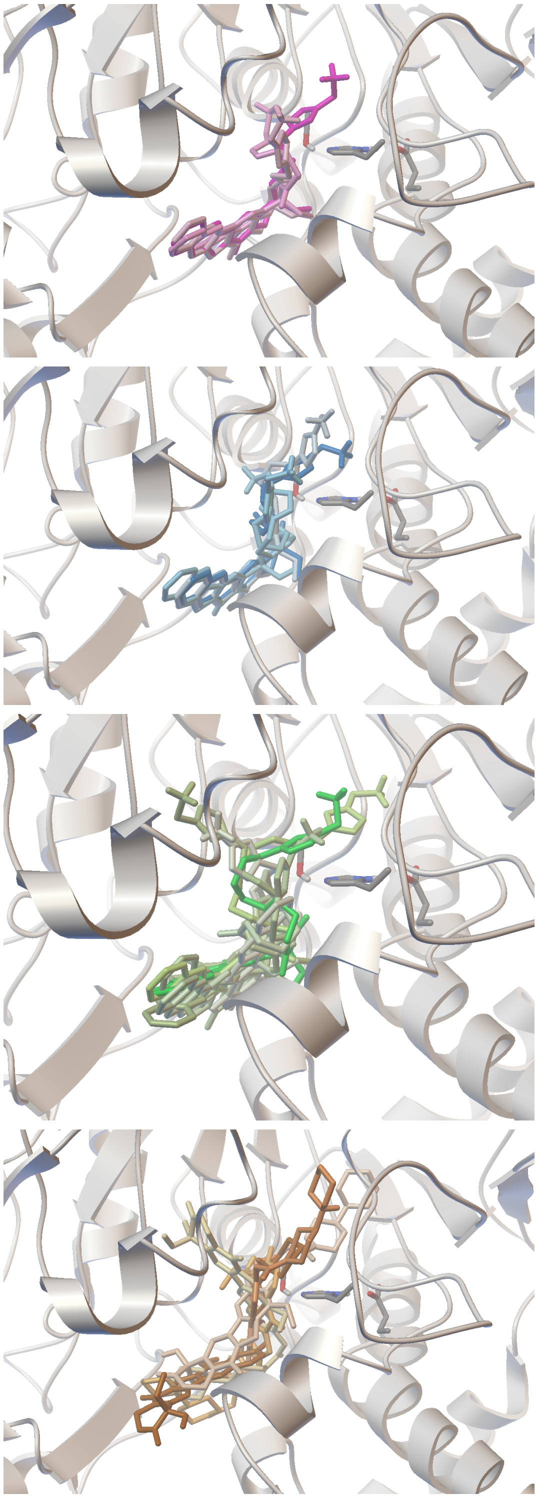

L2

L5

L8

Irinotecan

Figure S11: Docking poses of the ligands bound to HCE2. For clarity, only poses of the most populated clusters are shown. The poses of the different ligands subjected to molecular dynamics simulations are highlighted with darker colours.

L2 (SER–HID)

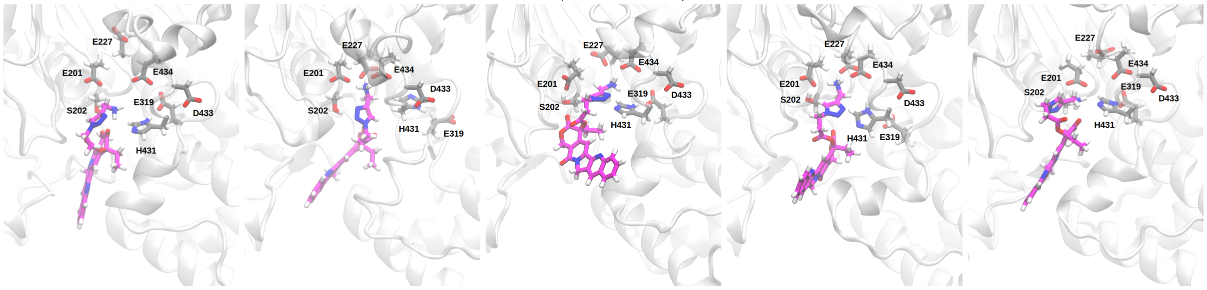

L5 (SER–HID)

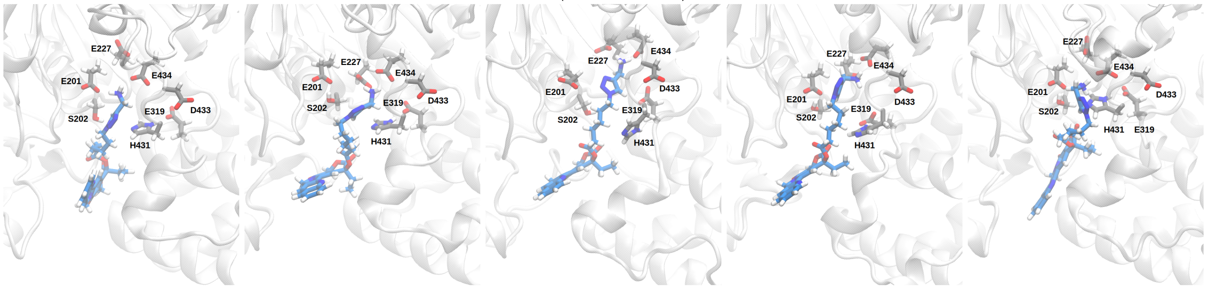

L8 (SER–HID)

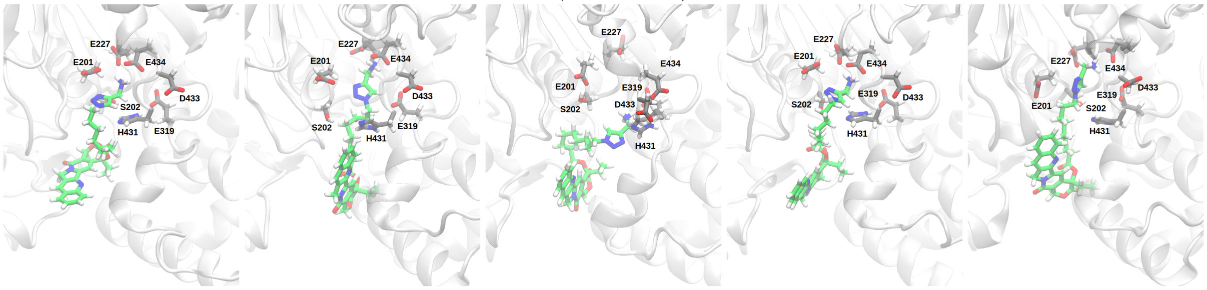

It (SER–HID)

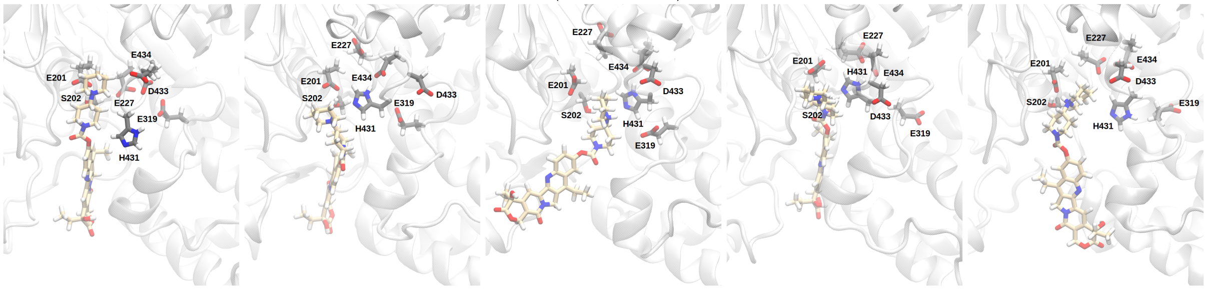

Figure S12: Snapshots (medoid structures) of the five individual MD simulations of all (SER–HID) models of the HCE2-ligand complexes.

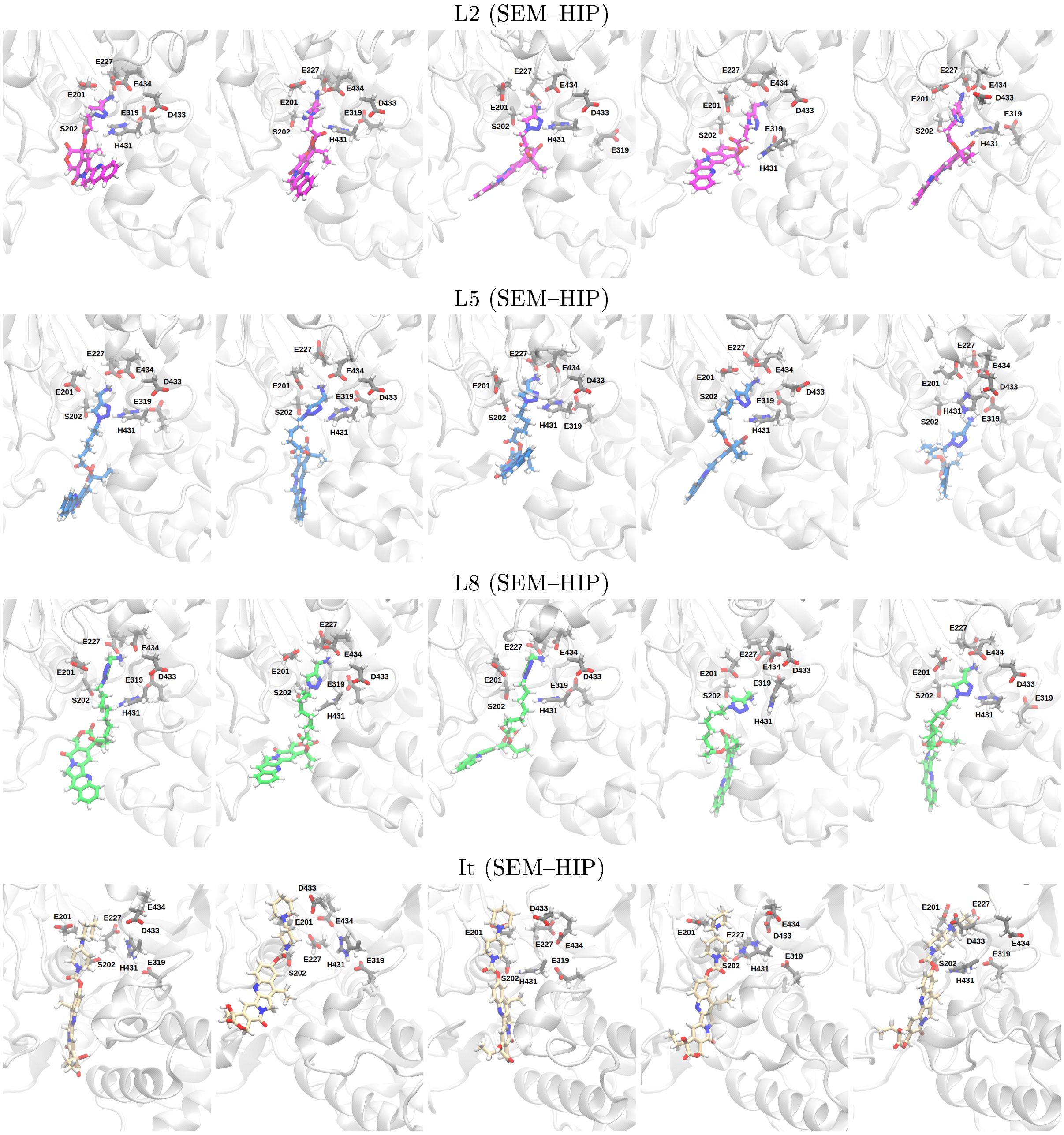

Figure S13: Snapshots (medoid structures) of the five individual MD simulations of all (SEM–HIP) models of the HCE2-ligand complexes.

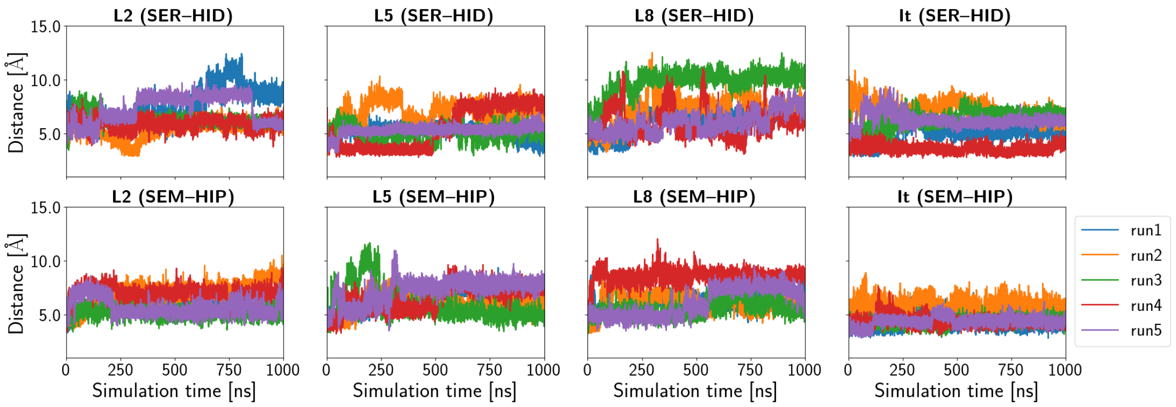

Figure S14: Time series of the distances between Ser202 and the ester carbonyl C-atom of the ligands.

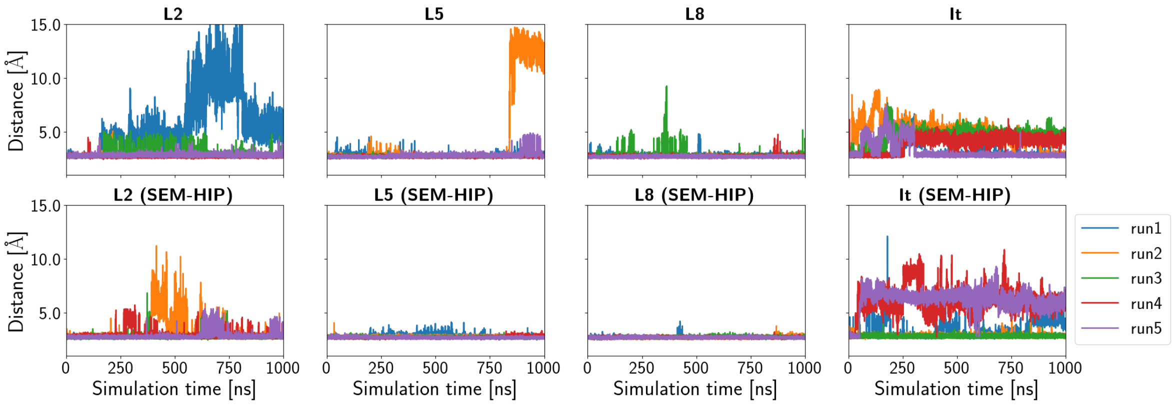

Figure S15: Time series of the distances between the closest carboxyl oxygen atom of a Glu or Asp residue and the amino N-atom of the ligands.

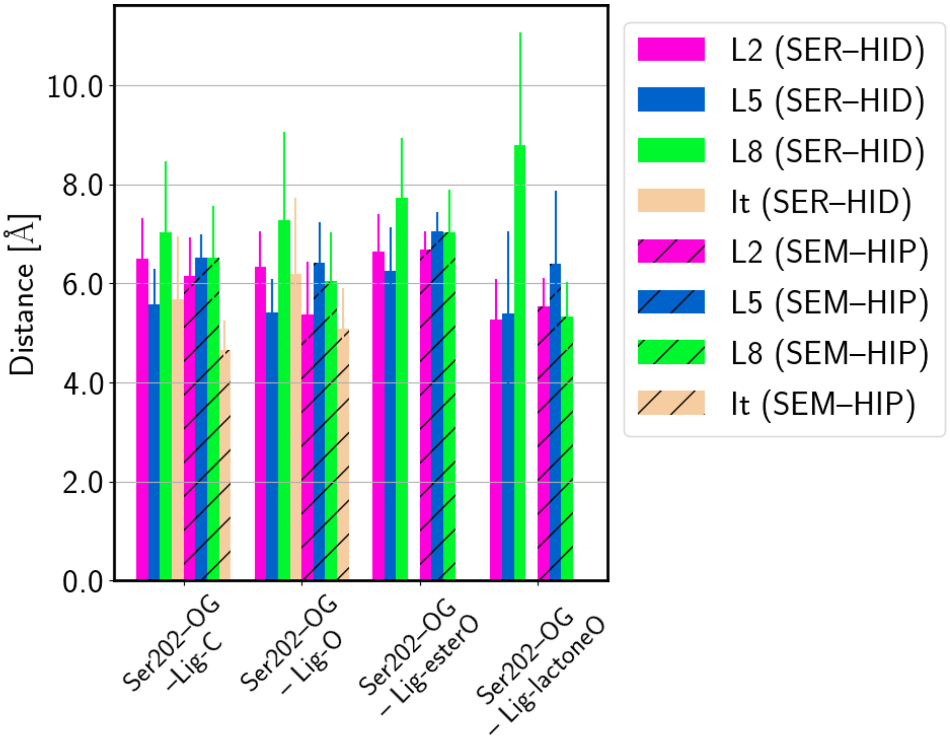

Figure S16: Average distance between the catalytic Ser202 and different atoms of the ligands.

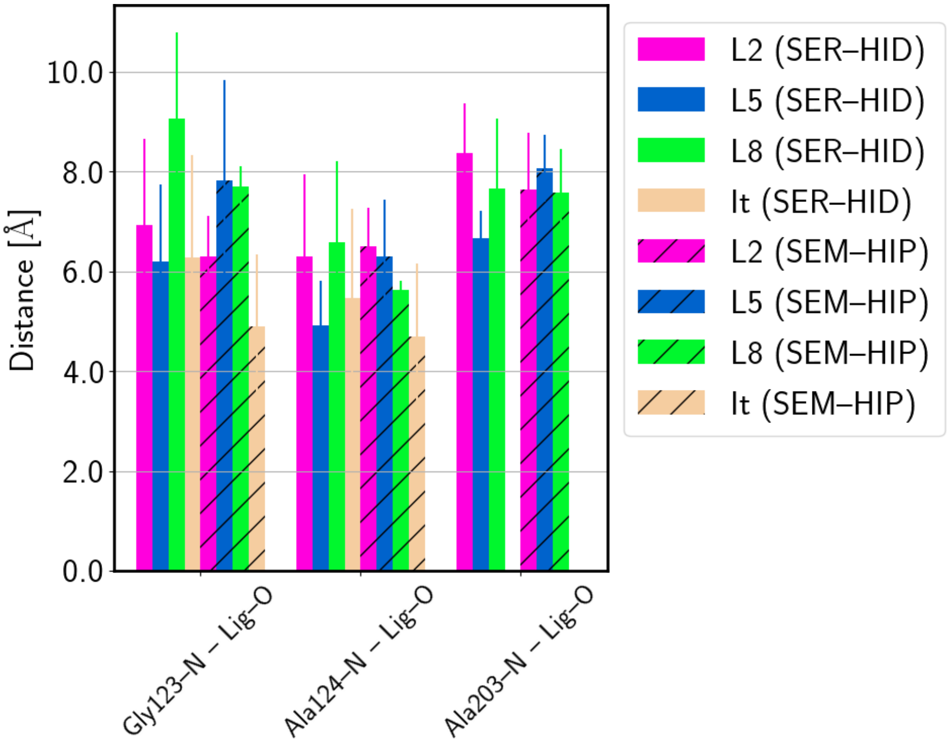

Figure S17: Average distances between residues considered as forming the "oxyanion hole", Gly123, Ala124, and Ala203, to the carbonyl oxygen atom.
